# GD2+ cancer stem cells in triple-negative breast cancer: mechanisms of resistance to breast cancer therapies

**DOI:** 10.20517/cdr.2022.30

**Published:** 2022-06-22

**Authors:** Khoa Nguyen, Emily McConnell, Orielle Edwards, Bridgette M. Collins-Burow, Matthew E. Burow

**Affiliations:** Department of Medicine, Tulane University School of Medicine, New Orleans, LA 70112, USA.

**Keywords:** Drug resistance, breast cancer therapy, triple-negative breast cancer, cancer stem cells

## Abstract

Research has led to the development of tailored treatment options for different cancers in different patients. Despite some treatments being able to provide remarkable responses, nearly all current treatments encounter the same issue: resistance. Here, we discuss our experiences with how breast cancers resist therapies. The focus of our discussion revolves around the cancer stem cell subpopulation and their mechanisms for resistance.

## INTRODUCTION

In 2021, breast cancer became the most common globally diagnosed cancer, accounting for 12% of all annual cases, with over 40,000 American women projected to die of breast cancer in 2022^[1]^. Despite advancing therapies, treatment resistance has been shown to be responsible for up to 90% of cancer-related deaths^[2,3]^. The purpose of this perspective is to share our group’s cumulative experiences in researching breast cancer and the mechanisms of treatment resistance. To do this, we discuss literature and findings from our group and others. The discussions focus on the triple-negative breast cancer (TNBC) subtype and the cancer stem cell (CSC) subpopulation. Furthermore, we will place emphasis on the CSC biomarker GD2 due to its novelty and integral role in our research. We hope that our text will promote discussions and encourage other research groups to incorporate our understanding and findings into their projects.

### Breast cancer subtypes: TNBC and hormone receptor therapies

Breast cancer is a complex malignancy that can be molecularly characterized into subtypes with unique disease progressions, treatment regimens, and prognoses^[4]^. Subtyping is based on the tumor’s hormone receptor status and can be grouped as follows: Luminal A (ER+, PR+, HER2-, KI67-), Luminal B (ER+, PR+, HER2±, KI67+), HER2 overexpression (ER-, PR-, HER2+), and Basal/Triple Negative (ER-, PR-, HER2-)^[5]^. Although TNBC can be further classified (basal-like 1, basal-like 2, immunomodulatory, mesenchymal, mesenchymal stem-like, and luminal androgen receptor), the overall characteristics are a highly invasive, metastatic, and recurrent breast cancer subtype with high mortality rates and few treatment options^[6,7]^. TNBC’s lack of receptor expression allows it to resist current hormone receptor therapies such as tamoxifen, aromatase inhibitors, luteinizing hormone-releasing hormone agonists, Faslodex, and trastuzumab^[8-10]^. As a result, current treatments are limited to conventional therapeutics such as tumor resection, chemotherapy, and radiation therapies^[11]^. Despite initial responses, TNBC patients have a higher rate of distant recurrence with significantly worse 3-year survival probabilities post-neoadjuvant treatment compared to other breast cancer subtypes^[12]^. Therefore, identifying specific therapeutic targets for TNBC is imperative for improving treatment outcomes for patients.

### Cancer stem cells: resisting chemo- and radiotherapies

Carcinogenesis, the transformation of healthy to cancerous cells, is due to an accumulation of mutations that results in uncontrolled replication and can often begin with a single cell. Despite their monoclonal origin, tumors are composed of a heterogeneous population of cells with only a small subpopulation possessing tumorigenic potential^[13]^. CSCs are a sub-subpopulation of tumors that possess the ability to self-renew, have increased tumorigenic potential, and increased mobility. Additionally, CSCs are innately resistant to radiotherapy and chemotherapies^[14-16]^. Although chemo- and radiotherapies may be initially successful in killing off most cells in a tumor, they confer a selection pressure for CSCs. This leads to the recurrence of a treatment-resistant tumor and an overall poorer outcome for TNBC patients in comparison to other breast cancer subtypes.

Chemotherapies and radiotherapy primarily affect rapidly dividing cells by interfering with DNA processing, leading to cellular apoptosis. Cancer stem cells have several mechanisms that allow them to resist and overcome the effects of chemotherapy. For example, CSCs have the ability to become quiescent in stressful conditions, hibernating in G_0_ and re-entering the cell cycle once conditions have improved. Malladi *et al.* identified SOX2 and SOX9 transcription factors producing DKK1 to inhibit WNT as the mechanism for quiescence in early-stage human lung and breast carcinoma cell lines^[17]^. This lack of cell division and, by association, DNA replication decreases the effectiveness of DNA targeting therapies such as chemo- and radiotherapy. In addition to becoming quiescent, CSCs can express ABC transporter proteins and efflux pumps that actively remove toxic compounds from within the cell. Das *et al.* were able to enhance the chemosensitivity of breast cancer stem cells by downregulating ABCG2 using nanoparticles^[18]^. Similarly, Sun *et al. *enhanced doxorubicin sensitivity, demonstrated by a 90% decrease in IC50, after 60% downregulation of ABCG2 was achieved by siRNA^[19]^. Although these studies demonstrate the significance of ABC transporters and efflux pumps in CSCs, it is important to note that varying levels of these proteins can be expressed in the non-stem population of the tumor as well.

Apoptosis is the natural cell death response to DNA damage. If quiescence and ABC transport proteins fail to prevent DNA damage from chemo- and radiotherapies, cancer cells will undergo apoptosis. This can be seen during the initial responsiveness of tumors to chemo- and radiotherapies. However, cancer stem cells have intrinsic mechanisms that allow them to avoid apoptosis. Takeda *et al.* identified a ROCK-survivin anti-apoptotic axis in pancreatic cancer stem cells and that inhibiting ROCK resulted in decreased survivin expression, leading to the sensitization of cells to gemcitabine^[20]^. Although there are more pathways associated with cancer stem cells and their resistance to therapies, such as Hippo/YAP1, Wnt/ß-catenin, Notch, and JAK/STAT, the main takeaway is that cancer stem cells can resist therapies through quiescence, drug efflux, and anti-apoptosis^[21]^. Overall, these mechanisms make CSCs difficult to target. When combined with hormone receptor therapy resistance, these qualities make TNBC an especially difficult disease to treat. Consequently, understanding the cellular biology of CSCs in TNBC, and other cancers, can provide insight into future novel therapies^[22,23]^.

### GD2: a cancer stem cell biomarker

Stem cells are essential for their abilities to self-renew and differentiate and are responsible for migrating to biological niches to replace damaged or depleted cell types^[24]^. In cancer, these same qualities lead to disease progression, metastasis, and patient mortality. It is important, then, to characterize CSC-specific cell surface markers for targeted treatment. In 2003, Al-Hajj *et al. *identified CD44high/CD24low as breast CSC markers^[25]^. Shortly after, ALDH1 was identified as another breast CSC marker, although primarily for luminal subtypes^[26]^. However, these markers are poor targets for therapy because they are also expressed by normal human breast cells^[27]^.

In 2012, Battula *et al. *identified GD2 as a novel breast cancer stem cell marker in TNBC and that it is co-expressed with CD44^high^/CD24^low^ cells^[28]^. GD2, a b-series ganglioside located within lipid rafts on the outer side of the plasma membrane, is expressed during development but is highly restricted to cerebellar tissues and peripheral nerves in mature adults. It can also be found on tumors of neuroectodermal origin such as neuroblastoma and melanoma^[29,30]^. Battula *et al. *and Jaggupilli *et al. *then went on to identify NF-kB signaling, an inflammatory pathway, and metabolic stress, respectively, as primary drivers of GD2 expression^[31,32]^. Further studies by Nguyen *et al.* identified GD2 as an activator of the FAK-AKT-mTOR signaling pathway, leading to increased tumor growth and metastasis in *in vivo* conditions^[33]^. Furthermore, this study demonstrated that CRISPR knock-out of GD3S, the rate-limiting enzyme for GD2 synthesis, completely ablated GD2 cells. Interestingly, GD3S KO cells resulted in no *in vivo *tumor growth and metastasis, greatly reduced *in vitro *migration, invasion, and growth in 3D conditions, but had no effect on *in vitro *2D proliferation. Patient studies have shown that high levels of GD2 have been associated with advanced cancer stage, larger tumor size, nodal invasion, and enhanced tumor proliferation and invasiveness^[34]^. These clinical correlations make GD2 an ideal breast CSC therapeutic target.

### Targeting GD2 to treat cancer stem cells

Characterized by its lack of hormone receptor status, patients diagnosed with TNBC are typically unresponsive to target-specific hormone receptor therapies. Treatment options primarily consist of surgery, neoadjuvant therapies, and chemotherapy with standard cytotoxic agents. Despite initial responses, the overall prognosis remains poor^[35,36]^. GD2 has been identified as a suitable therapeutic biomarker for targeting breast CSCs because of its limited expression in normal, healthy tissues. Because of this, therapies that target GD2 could be a promising avenue for treatment development. Battula *et al.* identified BMS-345541 as a potential upstream small molecule inhibitor for GD2 by blocking IKK activity, thus suppressing NF-kB signaling^[31]^. Although NF-kB is ubiquitous in normal tissues and cells, it is typically inactive until cellular stress responses such as inflammation occur. Furthermore, Jaggupilli *et al. *were able to reduce the GD2+ CSC population by 70%-80% using glutamine uptake inhibitor, V9302^[32]^. Lastly, Nguyen *et al*. were able to suppress the activity of GD2+ CSCs by inhibiting downstream activation of FAK and mTOR using PF-573228 and everolimus, respectively^[33]^.

In addition to targeting the upstream and downstream mechanisms of GD2 expression and activity, immune-based therapies that target GD2 expressing cells could also be pursued. Seitz *et al. *accomplished this by developing CAR T-cells (chimeric antigen receptor T-cells) that are genetically engineered to target GD2 expressing cells^[37]^. Although their studies demonstrated anti-GD2 CAR T-cells to be effective at reducing metastasis *in vivo*, graft-versus-host disease (GVHD) is a major cause of clinical morbidity and mortality^[38]^. To overcome the limitations associated with T-cells, natural killer (NK) cells could be generated instead. Esser *et al. *demonstrated that engineered GD2-specific NK cells were able to induce ADCC-like (antibody-dependent cellular cytotoxicity) killing effects against neuroectodermal tumors^[39]^. Furthermore, Ly *et al. *determined that infusion of anti-GD2 antibody dinutuximab, a chimeric human-mouse monoclonal antibody, could inhibit tumor growth *in vivo *and extend the survival of mice with TNBC via NK cell-mediated antibody-dependent cellular cytotoxicity^[34]^. The FDA has already approved GD2-specific therapies for the treatment of neuroblastoma, osteosarcoma, and other GD2+ cancers. Additionally, other anti-GD2 therapies, such as naxitamab and an anti-GD2-GD3 vaccine, have been granted FDA approval^[40,41]^. Anti-GD2 antibodies, however, do confer toxicity and are associated with adverse side effects including neuropathy, fever, significant pain, and allergies^[42]^. Neuropathic pain is likely due to GD2 expression in neural cells; however, *O-*acetyl-GD2 (OAcGD2), a GD2 derivative, is expressed on GD2+ solid tumor cells but not on neural fibers, and specific targeting OAcGD2 may reduce the aforementioned side effects^[43]^. Overall, these compounds may be beneficial for the treatment of TNBC, in combination with other therapies, by targeting the CSC subpopulation.

## SUMMARY

Breast cancer is now the most commonly diagnosed cancer, accounting for 12% of global cases. Although advances in breast cancer treatment have been made, treatment resistance remains a problem for many patients. This is especially true for patients diagnosed with triple-negative breast cancer because of this cancer subtype’s inherent ability to resist hormone receptor targeted therapies. Additionally, treatment with chemo- and radiotherapies is selected for the cancer stem cell subpopulation, a subset of cancer cells that is resistant due to mechanisms involving quiescence, DNA replication and repair, and apoptosis. Furthermore, these CSCs have an increased tumorigenic and metastatic potential.

GD2 has been identified as a breast cancer stem cell marker and is a promising target for breast cancer therapy^[44]^. Furthermore, dinutuximab is a GD2-specific monoclonal antibody that is FDA approved for neuroblastoma. Because of this, we believe incorporating methods for targeting CSCs, specifically by GD2, into current TNBC research may lead to promising results. Examples of this would be GD2 combination therapies with novel pathway inhibitors, analyzing the effects in the tumor microenvironment, and studying the role of CSCs on the tumor architecture by looking at the extracellular matrix. Any discoveries could potentially lead to rapid clinical turnarounds due to the FDA-approved status of dinutuximab.

## DECLARATIONS

### Copyright

© The Author(s) 2022.
